# The Postal Service and the Medical Press, with Reflections

**Published:** 1907-01

**Authors:** 


### THE POSTAL SERVICE AND THE MEDICAL PRESS, WITH REFLECTIONS.

"I will confess this. If I were running the Post Office service as a private institution, it would not cost me so much."

The words quoted above are part of a reply by Mr. Edwin C. Maddon, third assistant postmaster general, the author of the scheme to quadruple the cost of mailing on newspapers, to a question put to him before the Postal Commission appointed by Congress to investigate the conditions relative to the postal deficit, which, it is alleged, is caused by the inadequacy of the present rates for second class matter.

A curious commentary on, and confirmation of, the principle involved as a corollary in Mr. Madden's statement comes in a proposition made by Mr. W. D. Boyce, of Chicago, multi-millionaire, publisher, man of affairs, and president of the American Weekly Publishers' Association. According to the *Washington Times* for November 27th, Mr. Boyce, with the aid of other capitalists, has offered to take over the entire post office business of the country on the following terms: A private corporation to be formed, having a $50,000,000 capital and operating under full government regulation; *all postal rates to be forthwith reduced one-half;* the public postal buildings or quarters in public buildings to be rented by the corporation from the government; the ogvernment to be charged regular rates for all services rendered to it, which, under $25,000,000 to $12,500,000; all sinecures and ornamental appointments to be abolished, and postal matters to be entirely removed from political influence; and finally, the corporation to hand over to the government all profits that may accrue in excess of 7 per cent.

And how is it proposed that this shall be done? Simply by putting into practice the hint given in Mr. Madden's remark, and applying business principles and enterprise to the postal service, including its development in new directions for the public convenience. As one instance in point, Mr. Boyce states that the projectors have a plan for the establishment of a rural express, whereby rural carriers should receive and deliver goods on their routes, carrying the packages outside the mail.

It is somewhat significant that in these days, when the gathering storm is heard in the distance clamoring to transfer all the "public utilities" from private to government control, and when in support of such a course its success is delcared to have been demonstrated by the railways in New Zealond, the street car system in Glasgow, and many similar successful experiments, a reverse current should set in in the United States, as a consequence of the confessed inability of the government to make the postal service pay, while a private corporation stands prepared to undertake the task under government control at half the present cost both to the users of the service and to the government. Perhaps this fact explains the reluctance of many far sighted men of affairs in this country to fall in with nationalization and municipalization plans—the knowledge that sources of leakage consequent on the jobbery of our political system exist, the absence of which in other countries renders the success of such enterprises as government departments a possibility.

At any rate, it seems to us, that it is "up to" the government, if it wishes to sustain the boast of being a government of the people, by the people, for the people, either itself to accomplish the needed retrenchment in a fair and honorable manner, and not by a piece of flagrant iniquity, or to take up Mr. Boyce's challenge, and let him and his associates try their hands under its supervision.

